# Cement Dust Exposure and Perturbations in Some Elements and Lung and Liver Functions of Cement Factory Workers

**DOI:** 10.1155/2016/6104719

**Published:** 2016-02-11

**Authors:** Egbe Edmund Richard, Nsonwu-Anyanwu Augusta Chinyere, Offor Sunday Jeremaiah, Usoro Chinyere Adanna Opara, Etukudo Maise Henrieta, Egbe Deborah Ifunanya

**Affiliations:** Department of Medical Laboratory Science, Faculty of Allied Medical Sciences, College of Medical Sciences, University of Calabar, Calabar 543000, Nigeria

## Abstract

*Background*. Cement dust inhalation is associated with deleterious health effects. The impact of cement dust exposure on the peak expiratory flow rate (PEFR), liver function, and some serum elements in workers and residents near cement factory were assessed.* Methods*. Two hundred and ten subjects (50 workers, 60 residents, and 100 controls) aged 18–60 years were studied. PEFR, liver function {aspartate and alanine transaminases (AST and ALT) and total and conjugated bilirubin (TB and CB)}, and serum elements {lead (Pb), copper (Cu), manganese (Mn), iron (Fe), cadmium (Cd), selenium (Se), chromium (Cr), zinc (Zn), and arsenic (As)} were determined using peak flow meter, colorimetry, and atomic absorption spectrometry, respectively. Data were analysed using ANOVA and correlation at *p* = 0.05.* Results*. The ALT, TB, CB, Pb, As, Cd, Cr, Se, Mn, and Cu were significantly higher and PEFR, Fe, and Zn lower in workers and residents compared to controls (*p* < 0.05). Higher levels of ALT, AST, and Fe and lower levels of Pb, Cd, Cr, Se, Mn, and Cu were seen in cement workers compared to residents (*p* < 0.05). Negative correlation was observed between duration of exposure and PEFR (*r* = −0.416, *p* = 0.016) in cement workers.* Conclusions*. Cement dust inhalation may be associated with alterations in serum elements levels and lung and liver functions while long term exposure lowers peak expiratory flow rate.

## 1. Introduction

Deleterious effects of exposure to constituents of cement dust on organ system in humans have been described. Molecules of primary importance in cement dust in terms of content and potential health effects basically include 60–67% calcium oxide, 17–25 silicon oxide (SiO_2_), and 3–5% aluminium (Al) oxide, with some amount of iron oxide, chromium (Cr), potassium, sodium, sulphur, and magnesium oxide [1, 2]. Occupational exposures to aluminum (Al), iron (Fe), calcium (Ca), and silicon (Si) have been associated with decreased lung function indicators in exposed workers [3, 4]. Hepatosplenic silicosis and hepatic porphyria, with associated changes in liver function parameters, have also been described as some of the systemic effects of silica. Lipid peroxidation, oxidative damage, and immunologic mechanisms have been described as pathologic mechanisms of cement dust induced toxicities [5, 6]. Some essential elements necessary for normal physiologic functions are also constituents of cement dust. Toxicity from these essential trace elements occurs only when the exposure is above the range which can be accommodated by homeostatic mechanisms [7].

Studies on effects of cement dust exposure on lung and liver functions in occupationally exposed individuals in Nigeria have been documented [2, 8, 9]; however, information on the levels of some essential and nonessential elements and their relative or absolute contribution to lung and liver toxicities among individuals occupationally exposed to cement dust is still uncertain. This study therefore estimates the lung and liver functions and levels of some essential and nonessential elements in residents and workers in cement factory sites to determine their possible contribution to impairment of lung and liver functions in these individuals.

## 2. Materials and Methods

### 2.1. Study Design

This study was conducted at the United Cement Company (UNICEM) at Mfamosing, Akamkpa local government area, Cross River State, Nigeria. The Mfamosing limestone deposit serves as the major source of raw materials used by UNICEM for the production of ordinary Portland cement (OPC). Since July 2009, the Mfamosing cement plant produces approximately 2.5 million metric tons of cement per annum and currently employs about 350 permanent workers. The factory has four major departments: Production (Crusher, Raw Mill, Kiln, Cement Mill, and Packing Section), Engineering and Maintenance (Mechanical and Electrical Section), Mining, and Administration (Cashier, Administrative Officer, Security, and Marketing Section). The site is close to 200 m west to Mbebui village at coordinates 05.04493°N, 008.298995°E, 500 m south to Abifan community at coordinates 05.07591°N, 008.52192°E, and 200 m east to Mfamosing community and 100 m east to main quarry site at coordinates 05.06993°N, 008.53908°E [10].

### 2.2. Selection of Subjects

Two hundred and ten male subjects aged 18–60 years who fulfilled the inclusion criteria were randomly selected for this study. This study population comprised fifty regular cement factory workers, sixty residents of the communities surrounding the UNICEM factory, and one hundred apparently healthy individuals residing in Calabar metropolis 45 km away from the factory, not exposed to cement dust, who served as control subjects. Informed consent was obtained from them and ethical approval was obtained from The Center of Clinical Governance, Research and Training, Ministry of Health, Cross River State. This study was carried out in accordance with the Ethical Principles for Medical Research Involving Human Subjects as outlined in the Helsinki Declaration in 1975 (revised in 2000). Test subjects of the study were selected from the production section of the cement factory. This comprises permanent employees of the factory who are occupationally exposed to cement dust daily for not less than 2 years in the course of their work. Host community resident subjects were also individuals that had been resident in the communities proximal to the cement factory for not less than 2 years, while control subjects were individuals who had never been occupationally exposed to cement dust and are resident in Calabar metropolis.

Participants were notified several days before the commencement of the study and were given appropriate instructions. The physical characteristics of the subjects included weight and height measured with the use of weighing scale and stadiometer, respectively. Systolic and diastolic blood pressure were taken using sphygmomanometer. All subjects of the study were interviewed to establish their level of literacy and were assisted in filling the questionnaire to minimize errors. Sociodemographic data were collected by an interviewer-administered structured questionnaire aiming to determine age, educational levels, socioeconomic status, years of exposure as deduced from date of employment, site or position at the workplace, and use and nonuse of safety gadgets such as dust masks. Information on general health and history of past disease(s) and habits such as smoking, consumption of alcoholic beverages, and addictions were collected according to the British Medical Research Council questionnaire (BMRC, 1960). Individuals with a history of cigarette smoking, tobacco sniffing or chewing, liver disease or pulmonary disorders, chronic organ or systemic illness, and long term medication were excluded from the study.

### 2.3. Sample Collection

Blood samples were collected from midmorning to noon for both subjects and controls. Seven milliliters of blood was collected by venipuncture under aseptic conditions into a dry, clean plain sample container. The blood was allowed to clot and was centrifuged at 3,500 revolutions per minute for 5 minutes. After centrifuging, the serum was separated with the aid of a Pasture pipette and dispensed into dry chemically clean serum container, after which the samples were analysed immediately or stored at −20°C for subsequent analysis.

### 2.4. Laboratory Methods

#### 2.4.1. Estimation of Alanine Aminotransferase (ALT) [11]

Consider

(1) $$\begin{array}{c} \;\text{-}oxogutarate+L\text{-}alanine\overset{ALT}{\to }L\text{-}glutarate+pyruvate \end{array}$$

Alanine aminotransferase catalyzes the transfer of amino group from L-alanine to *α*-ketoglutarate forming pyruvate and L-glutamate. Pyruvate reacts with 2,4-dinitrophenylhydrazine to form 2,4-dinitrophenylhydrazone whose concentration is proportional to the ALT activity.

#### 2.4.2. Estimation of Aspartate Aminotransferase (AST), Reitman and Frankel [11]

Consider

(2) -oxoglutarate+L-aspartate→ASTglutamate+oxaloacetate

Aspartate aminotransferase (AST) catalyses the transfer of amino acid group from aspartate to ketoglutarate, forming oxaloacetate and glutamate. The oxaloacetate reacts with 2,4-dinitrophenylhydrazine to form 2,4-dinitrophenylhydrazone which in alkaline pH is reddish brown and whose concentration is proportional to the AST activity.

#### 2.4.3. Estimation of Bilirubin by Modified Valley's Method [12]

Serum bilirubin is present in two forms: conjugated which is mostly with glucuronic acid and unconjugated which is known as free bilirubin. Both react with diazotized sulphanilic acid to give a rose-purple azobilirubin. Conjugated bilirubin reacts in aqueous solution (direct reaction) whereas the unconjugated bilirubin requires an accelerator or solubilizer such as benzoate urea as in this method or alcohol which is used in other methods (indirect reaction).

#### 2.4.4. Estimation of Trace Element by Atomic Absorption Spectrophotometer [13]

An extract of the physiological sample is deproteinized and the filtrate is treated as lanthanum. This is aspirated in AAS which measures the absorbance of trace metals at various wavelengths corresponding to its bandwidth. The absorbance is proportional to the concentration of trace element in the sample.

#### 2.4.5. Estimation of Peak Expiratory Flow Rate by Peak Expiratory Flow Meter

The PEFR test is done with a peak expiratory flow meter: Spiroflow by Spirometrics, USA (originally described by Wright and McKerrow [14]). This is a simple handheld instrument with a mouthpiece on one end and a scale on the other. A small plastic arrow moves when air is blown into the mouthpiece, measuring the airflow speed. The subject breathes in deeply to full lung saturation and then blows into the mouthpiece as quickly and as hard as possible. The reading is taken and the procedure repeated two additional times. The highest record of the three is noted. If the subject coughs or sneezes while breathing out, the procedure will be repeated again. The PEFR readings were taken during break hour of 12 noon to 1 pm for the cement workers and for other subjects of the study.

### 2.5. Statistical Analysis

Data analysis was done using the statistical package for social sciences (SPSS version 20.0). Student's *t*-test analysis was used to determine mean differences between variables. Analysis of variance (ANOVA) was used to test significance of variations within and among group means and Fisher's least significant difference (LSD) post hoc test was used for comparism of multiple group means. Pearson correlation analysis was employed to determine relationship between variables. A two-sided probability value *p* < 0.05 was considered statistically significant

## 3. Results

Mean age, anthropometric indices (weight, height, and body mass index), systolic and diastolic blood pressure, some liver function tests (ALT, AST, TB, and CB), peak expiratory flow (PEFR), and serum elements levels (Fe, Zn, Pb, As, Cd, Cr, Se, Mn, and Cu) in cement workers, residents near the cement factory, and controls were shown in Table 1. Significant variations (*p* < 0.05) were observed in liver function test, peak expiratory flow, and serum elements levels in cement workers, residents living near the cement factory, and controls. No variations were observed in the levels of other indices studied (*p* > 0.05).

Table 2 shows comparison of some liver function test and PEFR of cement workers, residents near cement factory, and controls using post hoc analysis. The ALT, AST, TB, and CB were significantly higher and PEFR was lower in cement workers and residents near cement factory compared to controls (*p* < 0.05). The ALT and AST levels were significantly higher in cement workers compared to residents (*p* < 0.05). No significant differences were observed in TB, CB, and PEF levels in cement workers and residents (*p* > 0.05).

Comparison of serum elements levels in cement workers, residents near the cement factory, and controls using post hoc analysis was shown in Table 3. Cement workers and residents have significantly higher levels of Pb, As, Cd, Cr, Se, Mn, and Cu and lower levels of Fe and Zn compared to the controls (*p* < 0.05). Higher levels of Fe and lower levels of Pb, Cd, Cr, Se, Mn, and Cu were seen in cement workers compared to residents (*p* > 0.05). No significant differences were seen in Zn and As levels of cement workers and residents (*p* > 0.05).

**Figure 1:**
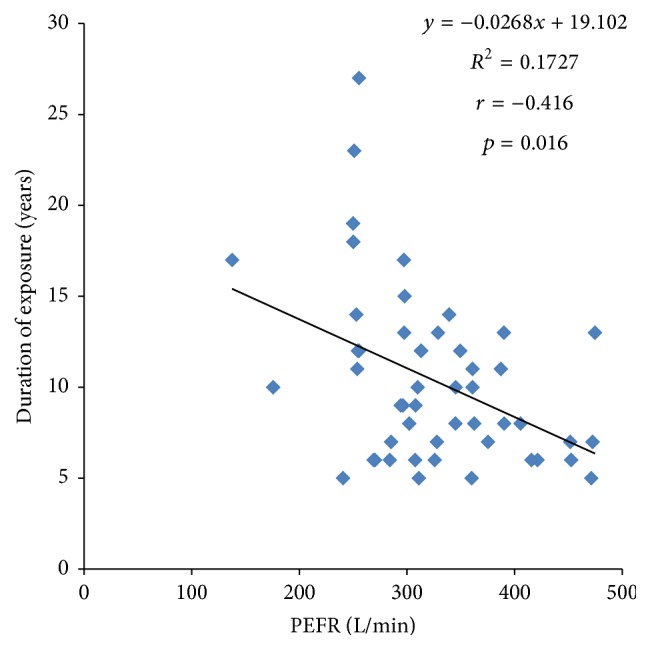
Correlation plot of duration of exposure to cement dust against peak expiratory flow rate in cement workers.

Figure 1 shows the correlation plot of duration of exposure to cement dust against peak expiratory flow rate in cement workers. A significant negative correlation (*r* = −0.416, *p* = 0.001) was observed between duration of exposure and peak expiratory flow rate in cement workers.

## 4. Discussion

The lung and liver function and some serum elements levels in occupationally exposed cement workers and residents near cement factories were studied. Decreased peak expiratory flow rate (PEFR) was observed in cement workers and residents living near cement factory when compared to unexposed controls. Similar findings have also been reported in cement workers in other developing countries [4, 15, 16]. Association of cement dust exposure with chronic impairment of lung function and respiratory symptoms such as cough, phlegm, and chest tightness and lung function indices has been described [17]. Accumulation of cement dust particles on upper and lower airways of the trachea-bronchial region of the lungs results in shortness of breath, chest tightness, sneezing, and coughing. Interaction between cement dust particles and the mast cell or basophil surface results in their degranulation and release of a variety of pharmacological active agents, including histamine and serotonin; the effect of these amines on tissues such as bronchial smooth muscles and vascular endothelium produces many of the symptoms of atopic conditions observed [18].

Prolonged duration of exposure to cement dust was associated with decreased peak expiratory flow. Similar observations have been reported by other studies [15, 16, 19, 20]. Bioaccumulation of some specific components as chromium and silica present in the cement dust in the respiratory tract may lead to delayed hypersensitivity reaction and chronic inflammation and hence impaired respiratory function. Contrary to our findings, a study by Fell et al. [1] reported no association between duration of cement dust exposure and decreased peak expiratory flow.

Increased levels of serum alanine aminotransferase, aspartate aminotransferase, and total and conjugated bilirubin were observed in cement workers compared to controls. The higher levels of aminotransferase observed in the cement workers are still within normal range for the liver enzyme in circulation and hence are not indicative of liver impairment. The liver has a high functional reserve capacity and only shows dysfunction when about 50% of the hepatocytes are affected. However, this increase may be an indication of potential for future development of hepatotoxic complications in occupationally exposed individuals. Our findings are in agreement with reports from other studies [8, 21]. Contrary to our findings, elevated serum levels of alanine aminotransferase, aspartate aminotransferase, and alkaline phosphatase above the normal ranges have been reported in cement workers by Al Salhen [22]. Cement dust inclusion particles, diffuse swelling, proliferation of hepatic sinusoidal lining cells, sarcoid granulomas, and perisinusoidal and portal fibrosis have been described in the hepatocytes of cement mill workers. These changes have been associated with hepatic lesions produced by inhaled cement dust [23].

Increased serum lead, arsenic, cadmium, chromium, selenium, manganese, and copper levels and lower iron and zinc levels were observed in cement workers and residents living near cement factory when compared to unexposed controls. Increased levels of chromium, copper, manganese, and selenium have also been demonstrated in cement factory workers and had been attributed to their exposure to cement dust [6, 24]. The toxic effect of most elements depends principally on the absorption, concentration, and persistence of the element at its site of action. These elements react with the endogenous target molecule such as receptors, enzymes, DNA, proteins, and lipids and critically alter their biologic functions, producing structural and functional changes that result in toxic damage [25]. Zinc and copper are components of antioxidant enzyme copper-zinc superoxide dismutase (Cu-Zn SOD). The increased demand on the antioxidant system to buffer the deleterious effects of heavy metal accumulation may account for lower zinc levels and a compensatory increase in copper levels seen in cement factory workers compared to unexposed controls. The body tends to retain copper to combat heavy antioxidant demands [6]. Decreased iron levels seen in cement workers may be associated with increased copper levels. Copper is a component of ceruloplasmin which catalyzes the oxidation of iron to ferric forms for binding to its transport protein transferrin and subsequent storage in tissues and synthesis of haemoglobin. Therefore, increased copper implies increased conversion and removal of circulating iron and hence decreased serum levels seen in cement factory workers [6].

Cement workers had higher levels of ALT, AST, and Fe and lower levels of Pb, Cd, Cr, Se, Mn, and Cu compared to residents of communities near the cement factory. The observation of higher serum levels of ALT, AST, and Fe and lower Cu seen has been discussed above. Contrary to our findings, no significant difference was reported in serum blood lead levels of cement workers and residents of neighboring community in a cement factory [26]. Lower levels of Pb, Cd, Cr, Se, and Mn in cement workers compared to residents may be attributed to the observation that cement factory workers are mandated to observe the laid down protective and safety precaution of the use of dust masks at factory site. Residents, on the other hand, do not observe such precautionary measures. Higher elements levels in residents compared to cement workers may also be attributed to duration of exposure which is also a determining factor in serum elements concentration. Cement workers are mere employees of the cement factory whose exposure to cement dust is dependent on duration of employment. Residents on the other hand spend almost all their life time in their community and are therefore exposed to cement dust on daily basis. The continuous inhalation or ingestion makes even the smallest concentration of such toxic elements a concern to their health. This is because the effects of exposure to any hazardous substance depend on the route of the exposure, concentration of substance, and duration of exposure. The level of toxicity found in short term exposure may be remedied, but the long term toxicity is associated with undesirable health consequences.

The findings of this study have shown that occupational exposure to cement dust may be associated with changes in the homeostasis of some essential and toxic elements resulting in higher levels of Pb, As, Cd, Cr, Se, Mn, and Cu and lower levels of Fe and Zn which may be implicated in impaired peak expiratory flow rate with potentials for future development of hepatotoxicity. These observations emphasize the need for adequate safety and precautionary measures among cement factory workers.

## Figures and Tables

**Table 1:** Mean age, anthropometric indices (weight, height, and BMI), systolic BP, diastolic BP, some liver function tests, peak expiratory flow rate, and serum elements levels in cement workers, residents near cement factory, and controls.

Parameter	Cement workers *n* = 50	Residents *n* = 60	Controls *n* = 100	*F*-value	*p* value
Age (years)	39.98 ± 5.56	38.32 ± 4.03	37.67 ± 5.32	0.453	0.636
Weight (kg)	61.04 ± 13.53	62.04 ± 12.55	61.90 ± 13.35	0.204	0.816
Height (meters)	1.67 ± 0.10	1.68 ± 0.09	1.72 ± 0.06	0.055	0.747
BMI (kg/m^2^)	23.35 ± 2.56	23.95 ± 2.32	23.10 ± 2.11	2.574	0.079
Sys. BP (mmHg)	128.80 ± 8.80	127.80 ± 8.83	128.80 ± 7.86	0.166	0.847
Diast. BP (mmHg)	84.20 ± 13.10	82.33 ± 10.14	82.50 ± 10.76	0.476	0.622
ALT (IU)	29.78 ± 2.58	11.25 ± 0.96	8.72 ± 0.41	76.53	0.000^*∗*^
AST (IU)	37.00 ± 2.61	17.41 ± 1.70	9.46 ± 0.64	84.29	0.000^*∗*^
TB (*μ*mol/L)	18.26 ± 0.91	18.18 ± 0.81	13.82 ± 0.62	12.80	0.000^*∗*^
CB (*μ*mol/L)	7.57 ± 0.58	7.33 ± 0.52	5.84 ± 0.27	5.66	0.004^*∗*^
PEFR (L/min)	324.96 ± 10.40	340.25 ± 10.38	400.17 ± 9.10	16.97	0.000^*∗*^
Fe (*μ*g/dL)	102.08 ± 1.78	96.13 ± 1.43	150.99 ± 1.35	447.99	0.000^*∗*^
Zn (*μ*g/dL)	80.94 ± 2.01	78.25 ± 1.86	106.52 ± 0.70	147.64	0.000^*∗*^
Pb (*μ*g/dL)	15.93 ± 0.42	20.09 ± 0.64	10.33 ± 0.60	70.32	0.000^*∗*^
As (*μ*g/dL)	0.011 ± 0.002	0.011 ± 0.005	0.003 ± 0.002	183.45	0.000^*∗*^
Cd (*μ*g/dL)	0.042 ± 0.008	1.950 ± 0.212	0.022 ± 0.001	102.20	0.000^*∗*^
Cr (*μ*g/dL)	0.033 ± 0.003	1.60 ± 0.125	0.012 ± 0.000	200.39	0.000^*∗*^
Se (*μ*g/dL)	0.022 ± 0.004	1.75 ± 0.127	0.044 ± 0.015	217.479	0.000^*∗*^
Mn (*μ*g/L)	2.970 ± 0.074	3.346 ± 0.062	2.579 ± 0.038	56.101	0.000^*∗*^
Cu (*μ*g/dL)	216.64 ± 6.93	234.62 ± 7.16	173.06 ± 3.662	37.83	0.000^*∗*^

^*∗*^Significant at *p* < 0.05; Sys. BP = systolic blood pressure; Diast. BP = diastolic blood pressure.

**Table 2:** Comparison of some liver function tests and peak expiratory flow in cement workers, residents near cement factory, and controls using post hoc analysis.

Parameter	Groups		Mean diff.	*p* value
	Cem. workers	Controls		
	*n* = 50	*n* = 100		
ALT (IU)	29.78 ± 2.57	8.72 ± 0.41	21.06 ± 1.75	0.000^*∗*^
AST (IU)	37.00 ± 2.61	9.46 ± 0.64	27.540 ± 2.12	0.000^*∗*^
TB (*μ*mol/L)	18.26 ± 0.91	13.82 ± 0.62	4.44 ± 1.09	0.000^*∗*^
CB (*μ*mol/L)	7.57 ± 0.58	5.84 ± 0.27	1.73 ± 0.60	0.004^*∗*^
PEFR (L/min)	324.96 ± 10.40	400.17 ± 9.10	−75.21 ± 14.58	0.000^*∗*^

	Residents	Controls		
	*n* = 60	*n* = 100		

ALT (IU)	11.25 ± 0.96	8.72 ± 0.41	2.53 ± 0.55	0.000^*∗*^
AST (IU)	17.41 ± 1.70	9.46 ± 0.64	7.95.90 ± 2.00	0.000^*∗*^
TB (*μ*mol/L)	18.18 ± 0.81	13.82 ± 0.62	4.36 ± 0.18	0.000^*∗*^
CB (*μ*mol/L)	7.33 ± 0.52	5.84 ± 0.27	1.49 ± 0.26	0.004^*∗*^
PEFR (L/min)	340.25 ± 10.38	400.17 ± 9.10	−59.92 ± 0.13	0.000^*∗*^

	Cem. workers	Residents		
	*n* = 50	*n* = 60		

ALT (IU)	29.78 ± 2.58	11.25 ± 0.96	18.53 ± 1.94	0.000^*∗*^
AST (IU)	37.00 ± 2.61	17.41 ± 1.70	19.58 ± 2.35	0.000^*∗*^
TB (*μ*mol/L)	18.264 ± 0.91	18.18 ± 0.81	.081 ± 1.204	0.947
CB (*μ*mol/L)	7.57 ± 0.58	7.33 ± 0.52	.24 ± 0.67	0.723
PEFR (L/min)	324.96 ± 10.40	340.25 ± 10.38	−15.29 ± 16.12	0.344

^*∗*^Significant at *p* < 0.05.

**Table 3:** Comparison of serum elements level in cement workers, residents near cement factory, and controls using post hoc analysis.

Parameter	Groups		Mean diff.	*p* value
	Cem. workers	Controls		
	*n* = 50	*n* = 100		
Fe (*μ*g/dL)	102.08 ± 1.78	150.99 ± 1.35	−48.92 ± 2.19	0.000^*∗*^
Zn (*μ*g/dL)	80.95 ± 2.01	106.52 ± 0.70	−25.58 ± 1.97	0.000^*∗*^
Pb (*μ*g/dL)	15.93 ± 0.42	10.33 ± 0.60	5.60 ± 0.89	0.000^*∗*^
As (*μ*g/dL)	0.011 ± 0.002	0.003 ± 0.002	0.008 ± 0.001	0.000^*∗*^
Cd (*μ*g/dL)	0.042 ± 0.008	0.022 ± 0.001	0.035 ± 0.005	0.000^*∗*^
Cr (*μ*g/dL)	0.033 ± 0.013	0.012 ± 0.000	0.021 ± 0.002	0.000^*∗*^
Se (*μ*g/dL)	0.023 ± 0.004	0.044 ± 0.015	−0.010 ± 0.002	0.000^*∗*^
Mn (*μ*g/L)	2.97 ± 0.074	2.58 ± 0.038	0.39 ± 0.078	0.000^*∗*^
Cu (*μ*g/dL)	216.65 ± 6.93	173.07 ± 3.66	43.58 ± 7.917	0.000^*∗*^

	Residents	Controls		
	*n* = 60	*n* = 100		

Fe (*μ*g/dL)	96.13 ± 1.429	150.99 ± 1.35	−54.86 ± 0.08	0.000^*∗*^
Zn (*μ*g/dL)	78.25 ± 1.863	106.52 ± 0.70	−28.28 ± 1.16	0.000^*∗*^
Pb (*μ*g/dL)	20.091 ± 0.643	10.33 ± 0.60	9.76 ± 0.04	0.000^*∗*^
As (*μ*g/dL)	0.011 ± 0.005	0.003 ± 0.002	0.007 ± 0.003	0.000^*∗*^
Cd (*μ*g/dL)	1.95 ± 0.21	0.022 ± 0.001	1.93 ± 0.143	0.000^*∗*^
Cr (*μ*g/dL)	1.60 ± 0.13	0.012 ± 0.000	1.60 ± 0.08	0.000^*∗*^
Se (*μ*g/dL)	1.75 ± 0.13	0.044 ± 0.015	1.71 ± 0.09	0.000^*∗*^
Mn (*μ*g/L)	3.35 ± 0.06	2.58 ± 0.038	0.77 ± 0.07	0.000^*∗*^
Cu (*μ*g/dL)	234.62 ± 7.17	173.07 ± 3.66	61.56 ± 0.50	0.000^*∗*^

	Cem. workers	Residents		
	*n* = 50	*n* = 60		

Fe (*μ*g/dL)	102.08 ± 1.78	96.13 ± 1.43	5.945 ± 2.422	0.015^*∗*^
Zn (*μ*g/dL)	80.95 ± 2.007	78.25 ± 1.86	2.69 ± 2.19	0.219
Pb (*μ*g/dL)	15.93 ± 0.42	20.09 ± 0.64	−4.16 ± 0.98	0.000^*∗*^
As (*μ*g/dL)	0.011 ± 0.002	0.011 ± 0.005	.0002 ± 0.0005	0.693
Cd (*μ*g/dL)	0.042 ± 0.008	1.95 ± 0.212	−1.91 ± 0.168	0.000^*∗*^
Cr (*μ*g/dL)	0.033 ± 0.013	1.60 ± 0.125	−1.57 ± 0.10	0.000^*∗*^
Se (*μ*g/dL)	0.023 ± 0.004	1.75 ± 0.127	−1.73 ± 0.103	0.000^*∗*^
Mn (*μ*g/L)	2.97 ± 0.074	3.35 ± 0.062	−.38 ± 0.09	0.000^*∗*^
Cu (*μ*g/dL)	216.65 ± 6.93	234.62 ± 7.17	−17.97 ± 8.75	0.041^*∗*^

^*∗*^Significant at *p* < 0.05.
